# 3D chromatin dynamics in cellular neuroendocrine transformation

**DOI:** 10.1093/procel/pwaf082

**Published:** 2025-10-17

**Authors:** Jianhua Xiong

**Affiliations:** Department of Urology, Emory University School of Medicine, Atlanta, GA 30322, United States; Winship Cancer Institute of Emory University, Atlanta, GA 30322, United States

Prostate cancer (PCa) cell fate has been a focal point of extensive research due to its high prevalence and the significant clinical challenges it poses (Siegel et al., 2025). In its advanced stages, castration-resistant prostate cancer (CRPC) frequently transitions into a neuroendocrine (NE) subtype, termed neuroendocrine prostate cancer (NEPC). This aggressive form is characterized by NE phenotypes, resistance to androgen receptor (AR) pathway inhibitors, and dismal clinical outcomes, with a median survival of less than one year after diagnosis (Laudato et al., 2019; Sargos et al., 2014). While considerable progress has been made in unraveling the molecular mechanisms underlying CRPC and NEPC (Laudato et al., 2019; Sargos et al., 2014; Siegel et al., 2025), the precise epigenetic and chromatin-level dynamics driving this cellular transformation have remained unclear. A recent study by Lu et al. has now revealed how coordinated epigenetic remodeling and three-dimensional (3D) genome reorganization drive NEPC progression, providing new insights into the regulatory networks that underpin cellular lineage plasticity and highlighting potential therapeutic opportunities (Lu et al., 2025).

## Unveiling 3D chromatin reorganization

The study takes a bold step in examining the 3D chromatin landscape of NEPC tumors compared to CRPC. Using high-throughput chromosome conformation capture analysis of patient-derived xenograft tumors, the authors revealed significant variations in the 3D chromatin structure between these two subtypes (Lu et al., 2025). This finding is significant because 3D chromatin architecture—the spatial organization of the genome within the nucleus—plays a critical role in regulating gene expression and maintaining cellular identity (Kim and Shendure, 2019; Rao et al., 2014). While earlier research emphasized the importance of epigenetic changes, such as DNA methylation and histone modifications, in NEPC development (Laudato et al., 2019), this study advances our understanding by highlighting chromatin reorganization as a key driver of this transformation.

**Figure 1.**
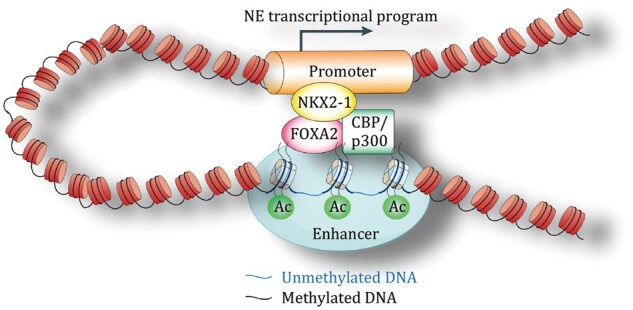
**FOXA2 and NKX2-1 cooperate to drive neuroendocrine prostate cancer (NEPC) transformation through 3D chromatin looping and epigenetic reprogramming**. FOXA2 acts as a pioneer factor that binds to previously inaccessible neuroendocrine (NE) lineage enhancers, induces regional DNA demethylation, and increases local chromatin accessibility. This enhancer activation leads to the transcriptional induction of NKX2-1, which, in turn, binds to gene promoters and interacts with FOXA2 at enhancer regions via chromatin looping. Together, FOXA2 and NKX2-1 form a self-reinforcing regulatory circuit that sustains NE lineage programs. They jointly recruit CBP/p300 coactivators, promoting NE enhancer activation through H3K27ac deposition and driving NEPC progression.

To further explore these dynamics, the researchers utilized isogenic prostate adenocarcinoma cells undergoing cellular neuroendocrine transformation (NET) as a model (Lu et al., 2025). This approach successfully recapitulated the chromatin architecture changes observed in NEPC, offering a robust system to investigate how these alterations enable the transcriptional reprogramming necessary for the transition from a luminal (AR-dependent) phenotype to a neuroendocrine (AR-independent) state. The results were compelling: the chromatin underwent significant reshaping, forming new interaction regions that were absent in CRPC models (Fig. 1).

## NKX2-1 and FOXA2: master regulators of NEPC

At the core of this chromatin transformation is the neural transcription factor NKX2-1, which is specifically and abundantly expressed in NEPC tumors. NKX2-1 is a homeodomain transcription factor that regulates neuronal progenitor identity and interneuron specification (Lu et al., 2025; Sandberg et al., 2016). Remarkably, its role in prostate cancer had not been well characterized before this study, making its identification in NEPC a significant discovery. Forkhead box A2 (FOXA2) functions as the pioneer factor that initiates neuroendocrine lineage reprogramming by binding to previously inaccessible enhancers, inducing DNA demethylation, and increasing local chromatin accessibility. This epigenetic priming activates downstream neuroendocrine lineage transcription factors, including NKX2-1. Once induced, NKX2-1 partners with FOXA2 to reinforce and stabilize the neuroendocrine transcriptional network. Together, they organize enhancer–promoter interactions and establish a chromatin architecture that drives neuroendocrine gene expression (Han et al., 2022; Lu et al., 2025). In this model, FOXA2 initiates the lineage transition, while NKX2-1 amplifies and sustains FOXA2’s activity at neuroendocrine regulatory elements, forming a self-reinforcing loop that maintains NEPC identity (Fig. 1).

This hierarchical network of transcription factors is indispensable for reprogramming the genome during NET, enabling cells to adopt a new identity as NEPC. Furthermore, NKX2-1 and FOXA2 recruit coactivators such as the CREB-binding protein (CBP)/E1A binding protein P300 (p300) complex, which catalyzes the acetylation of Histone H3 Lysine 27 (H3K27ac), a hallmark of active enhancers (Lu et al., 2025; Nicosia et al., 2023). Together, these factors form an intricate regulatory system, working in concert to reshape the chromatin landscape and drive the emergence of NEPC (Fig. 1). This discovery provides a comprehensive model of how transcription factors, chromatin-pioneering proteins, and coactivators synergize to induce NEPC.

## Implications for treatment and therapeutic targeting

These findings extend beyond basic science, offering new therapeutic avenues for NEPC (Lu et al., 2025; Swami et al., 2020). By uncovering the epigenetic and chromatin-driven mechanisms of NEPC, the study opens potential treatments targeting these processes. A promising prospect is the use of CBP/p300 inhibitors to disrupt the epigenetic network sustaining NEPC. The authors showed that inhibiting CBP/p300 blocked NE enhancer activation and effectively halted NEPC tumor growth *in vitro* and *in vivo*. Several CBP/p300 inhibitors, including CCS1477 (NCT03568656) and FT-7051 (NCT04575766), are currently being evaluated in early-phase clinical trials for advanced solid tumors and prostate cancer (Li et al., 2025; Mabe et al., 2024), suggesting that targeting CBP/p300 may represent a promising therapeutic strategy for the aggressive and treatment-resistant NEPC subtype.

In addition to CBP/p300, the study suggests targeting other epigenetic modifiers involved in chromatin reorganization—such as those regulating enhancer-promoter looping or DNA demethylation. This could lead to combination therapies that target multiple points in chromatin remodeling, potentially overcoming the limitations of single-agent treatments. The study also highlights the importance of individual cell analysis in understanding cancer heterogeneity. Using single-cell multiome analyses, the authors tracked NET in PCa cells, capturing individual cells with dynamic intermediate epigenetic and transcriptomic states. This approach helps differentiate clonal transformation from a single population switch during NEPC transformation.

## Broader impact on cell biology research

The implications of this work extend to fundamental principles of cell biology, particularly how chromatin architecture and epigenetic regulation govern cellular identity and plasticity. By interrogating the 3D organization of the genome during dynamic cellular state transitions, this study reveals how spatial chromatin remodeling orchestrates gene expression programs that enable cells to reconfigure their transcriptional and functional profiles (Laudato et al., 2019; Swami et al., 2020).

These findings highlight the 3D genome as a central regulatory layer in cellular decision-making, shaping processes such as lineage commitment, differentiation, and stress adaptation. Importantly, they position chromatin architecture not merely as a structural feature, but as a dynamic and instructive determinant of cellular behavior.

By mapping the molecular networks and epigenetic mechanisms underlying chromatin reorganization, this work establishes a framework for understanding how genome architecture integrates with signaling pathways and transcriptional circuits to drive cell state transitions. Such insights are broadly relevant across cell biology, offering conceptual advances for fields ranging from development and regeneration to disease mechanisms rooted in disrupted genome organization.
